# A prebill control plane for record integrity in inpatient artificial intelligence-enabled clinical documentation workflows

**DOI:** 10.3389/fdgth.2026.1910263

**Published:** 2026-10-08

**Authors:** Stephanie Smith, Hani Judeh

**Affiliations:** 1 Accuity Delivery Systems, LLC d/b/a Accuity, Mount Laurel, NJ, United States; 2 Icahn School of Medicine at Mount Sinai, New York, NY, United States

**Keywords:** ambient AI scribes, clinical documentation integrity, clinical informatics, generative AI, inpatient documentation, prebill review, record integrity, revenue cycle governance

## Abstract

Ambient artificial intelligence (AI) scribes and other electronic health record (EHR)-integrated generative AI tools are entering clinical documentation workflows because they offer measurable, if still modest, gains in documentation efficiency and clinician experience. At the same time, the medical record remains a high-consequence informatics object that supports clinical communication, continuity, documentation clarification, coding, payment, and downstream organizational reporting. As AI-generated content moves through drafting, summarization, clarification, and coding-adjacent workflows, the central challenge is no longer simply whether a model can generate fluent text, but whether health systems can preserve record integrity as documentation moves downstream. We examine this problem in United States inpatient claim-linked workflows. This Perspective proposes a prebill control plane as a candidate governance model for that problem. We describe three recurring modes of record-integrity failure that motivate it: unsupported addition, clinically meaningful loss, and propagation across encounters and systems. We then outline five operational components of a minimum viable control plane: physician-led governance and oversight, evidence visibility, change control, monitoring, and auditability. The framework is intended to support safer adoption by protecting patient safety, clinician safety, and record integrity under real-world inpatient and mid-revenue-cycle workflow pressure.

## Introduction

Ambient AI documentation tools are being adopted because they address a real operational problem. Current evidence suggests modest reductions in documentation burden and workflow friction, while also demonstrating persistent risks of factual error, omission, confabulation, and the structural limitations of clinician review as a safeguard (1–9). The inpatient evidence base is growing but remains uneven: hospital-only studies increasingly support feasibility for discharge-summary generation, hospital-course summarization, patient-facing discharge transformation, and coding assistance under human review, while live bedside inpatient ambient-scribe evidence and claim-linked production outcomes remain comparatively limited (10–12).

Although ambient scribes are the most studied and visible example, the record-integrity problem addressed here is broader: AI-enabled mid-revenue-cycle technologies increasingly shape the medical record through drafting, summarization, documentation nudges, query support, coding-adjacent interpretation, and emerging autonomous coding workflows. Adoption is moving ahead of the evidence; that gap is not an argument against governance but the central reason a practical control model is needed now.

The medical record is the primary communication layer through which clinicians hand off evolving patient stories across time, setting, and role, and the evidentiary substrate for payment and downstream reporting. CMS remains explicit that services billed to Medicare must be supported by the medical record (13). In this Perspective, record integrity refers to the accuracy, completeness, provenance, and contextual faithfulness of clinical documentation as it moves from encounter narrative to downstream coded representation.

This Perspective makes three contributions: first, it defines record-integrity risk in AI-enabled documentation as unsupported addition, clinically meaningful loss, and propagation; second, it identifies the prebill interval as a practical control point where documentation, clarification, coding translation, and accountability converge; and third, it proposes a minimum viable control plane built on physician-led governance, evidence visibility, change control, monitoring, and auditability.

## A practical workflow problem

Consider an illustrative composite case. On a chaotic Friday night in an overwhelmed emergency department, a physician admits a 34-year-old woman with progressive shortness of breath and pleuritic chest pain after a long flight. She takes oral contraceptives. CT pulmonary angiography confirms a segmental pulmonary embolism.

The chart may still contain component signals—hypoxia, oxygen requirement, tachycardia, imaging findings, and provoking factors—but lose the narrative synthesis that connects them into a clinically meaningful story. That loss can occur when the emergency department note itself is generated as a compressed account—“Pulmonary embolism. Started on anticoagulation”—or when a richer emergency account is later summarized into the admission record. If the flattened narrative serves as a principal signal for CDI prioritization or downstream review, the encounter may not be selected even though supporting elements remain elsewhere in the chart. At prebill review, those elements may still be recoverable if the workflow makes them visible and reconnects them to the clinical story.

Nothing about the care was wrong. The failure is informational. The record remains superficially coherent while becoming less faithful to the encounter. AI shapes the medical record directly through drafting and summarization and indirectly through documentation nudges, prioritization signals, and coding-adjacent tools. In this case, compression weakens the coherent clinical signal on which downstream prioritization and review may depend, even though fragments of the underlying evidence remain in the chart. The next clinician to care for the patient may inherit only the flattened narrative.

## Three recurring modes of record-integrity failure

A practical way to understand AI-enabled documentation risk is to treat it as three recurring modes of record-integrity failure.

Unsupported addition occurs when generated documentation introduces diagnostic certainty or factual statements that exceed available clinical support. The harder problem goes beyond overt hallucination. Unsupported content can be plausible and embedded in otherwise acceptable text. In a comparative inpatient study, LLM-generated hospital medicine discharge narratives contained more errors and higher harmfulness scores than physician-generated narratives despite acceptable surface quality (4). In a separate emergency department documentation study, LLM-assisted drafts reduced writing time but still required revision and quality control (5). Recent benchmarking work also demonstrates that leading LLMs accept and elaborate on fabricated medical claims when those claims are wrapped in confident, clinically styled language, suggesting that the style of clinical writing can override the truth of its content (14). Surface quality cannot be assumed to equal fidelity (4, 5, 14).

Clinically meaningful loss is the reciprocal hazard. Record integrity can also degrade through compression. As in the composite scenario, qualifiers such as acuity, temporality, uncertainty, differential framing, and provoking context may disappear. In a medical record, these are not decorative details. They shape downstream interpretation by clinicians, documentation integrity professionals, coders, and reviewers. In practice, an AI-generated note can therefore be directionally correct while still becoming clinically thinner than the source encounter (3–5). Context and nuance provide defensibility to the inpatient admission and care delivered.

Propagation predates generative AI. Copy/paste, carry-forward, and inherited templates already allow erroneous content to persist across records and settings (15, 16). AI can increase the speed, scale, and opacity of propagation. Once embedded, weak or unsupported statements may influence later summaries, clarification patterns, coded outputs, and retrospective interpretations of the encounter. They may also affect data used by downstream models, although the clinical magnitude of this risk remains uncertain (17).

### Why prebill is a practical control interval

AHIMA's current CDI standards distinguish concurrent, prebill, and retrospective review and describe prebill review as occurring before claim submission (18). AHIMA and ACDIS also define the query as a communication tool used to clarify health-record documentation for documentation integrity and accurate code assignment (19). Together, these standards identify a defined operational interval in which the record can still be compared with recoverable source context before claim submission.

No single control point is optimal for every AI-related documentation risk. Controls should be placed where they can best prevent, detect, or correct the relevant failure. Point-of-care verification is preferable when AI output may affect immediate clinical decisions or when the originating clinician can efficiently correct the record, but it competes with clinical attention and cannot address changes introduced later. Upstream configuration controls are preferable for recurrent errors caused by model selection, prompt or template design, thresholds, or workflow placement, but cannot fully adjudicate patient-specific effects after downstream processes have acted. Retrospective audit is suited to detecting drift and low-frequency patterns, but may identify them only after the record or claim is difficult to correct. These controls are complementary rather than interchangeable.

Prebill has a distinct role in complex inpatient claim-linked workflows. It is the interval at which the cumulative effects of documentation drafting, summarization, documentation nudges, CDI prioritization, and coding-adjacent tools can be evaluated together in a nearly final record, while source context is often recoverable and correction remains possible before claim submission. This makes prebill particularly relevant to encounter-specific distortions not fully prevented upstream or detected at the point of care.

Prebill review is not the best investment in every setting. Low-complexity, low-variance workflows may be adequately governed through upstream validation and sampled retrospective audit. Prebill may also be a poor fit when the expected reduction in record-integrity risk does not justify added staffing, operational complexity, or claim latency. Organizations without established CDI, HIM, or coding-review capacity may need to begin with targeted review of high-risk workflows rather than a broad prebill layer. The claim is therefore bounded: where multiple record-shaping technologies converge in high-consequence inpatient claim-linked workflows, prebill offers a practical interval for integrated review, adjudication, and escalation. It does not replace controls elsewhere in the AI lifecycle.

## Existing controls are necessary but fragmented

Existing frameworks address adjacent parts of this problem. AHIMA and ACDIS govern CDI review and compliant query practice, now explicitly including technology-generated queries and nudges (18, 19); the Joint Commission and ECRI address copy/paste and copied-data safety (15, 16); NIST provides lifecycle risk-management guidance; HAIP structures vendor disclosure; and the Health & AI Policy Index maps the evolving policy landscape (20–22). Joint Commission–CHAI guidance reinforces governance, validation, monitoring, and workforce competency (23). Taken together, these sources do not specify an integrated governance layer at the prebill interval, where direct and indirect mechanisms of AI-shaped documentation converge before claim submission. A prebill control plane is intended to fill that operational gap.

### A minimum viable prebill control plane

Borrowing the term from network architecture, we use “control plane” to mean the governance layer that sets rules for how AI-shaped documentation is reviewed, routed, corrected, and audited. Although it is exercised most visibly at the prebill interval, after coding and before claim submission, the control plane is not a discrete workflow step; its rules apply across AI-enabled touchpoints from generation and clinician sign-off through CDI review and coding support (Figure 1). The model extends prospective CDI practice through five capabilities: physician-led governance and oversight, evidence visibility, change control, monitoring, and auditability. Table 1 summarizes each component, the risk it addresses, its operational mechanism, accountable function, and supporting standards, guidance, or evidence.

**Figure 1:**
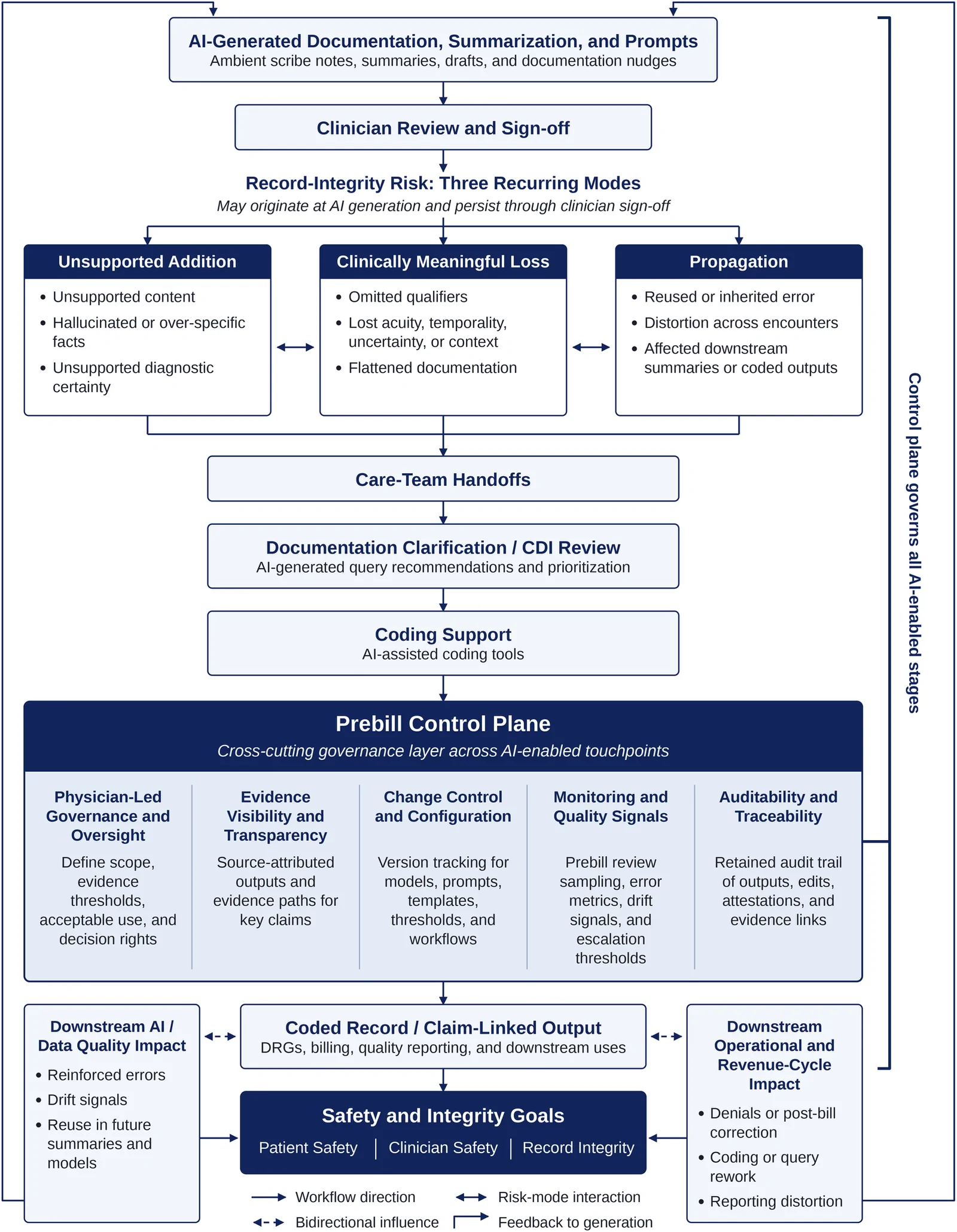
Prebill control plane for governing AI-enabled inpatient documentation workflows. AI-generated drafts and summaries, documentation prompts and nudges, prioritization signals, and coding-adjacent recommendations may shape the medical record both before clinician review and sign-off and as documentation moves through care-team handoffs, CDI review, coding support, and claim-linked workflows. Record-integrity risk may originate at AI generation and persist through clinician sign-off, arising through three recurring modes (unsupported addition, clinically meaningful loss, and propagation across encounters, summaries, coded outputs, and downstream systems) that can co-occur and compound as documentation moves downstream. The control plane is positioned at the prebill interval, after coding support and before claim-linked output, where these cumulative effects can be reviewed together in a nearly final record; the right-margin bracket indicates that it is a cross-cutting governance layer spanning AI-enabled touchpoints rather than a single review step. It comprises physician-led governance and oversight, evidence visibility and transparency, change control and configuration, monitoring and quality signals, and auditability and traceability. Downstream effects may include AI/data-quality feedback to upstream generation, operational and revenue-cycle consequences, and impacts on patient safety, clinician safety (including vigilance burden and liability exposure), and record integrity. Arrow types are defined in the figure key.

**Table 1:** Five components of a prebill control plane for record integrity in AI-enabled documentation workflows.

Component	Integrity risk addressed	Operational mechanism	Accountable function	Illustrative standard, guidance, or evidence source
Physician-led governance	Misalignment between AI scope and clinical meaning; unowned acceptance criteria	Standing physician-led governance group sets scope, acceptable-use criteria, and stop rules for AI tools affecting the legal record	Chief Medical Informatics Officer or equivalent; reports to medical staff and executive AI governance	NIST AI RMF Govern function (20); Joint Commission–CHAI Responsible Use of AI in Healthcare (2025) (23)
Evidence visibility	Unsupported addition; clinically meaningful loss; opaque provenance	Source-attributed AI outputs; evidence-linked review of factual claims against the patient's record before downstream acceptance or claim submission, especially for diagnoses and severity modifiers	Clinical informatics, supported by EHR and AI vendor	Chung et al., *NEJM AI* 2025 (6); AHIMA *Prospective Clinical Documentation Integrity (CDI) Reviews and Query/Alert Practice Best Standards* (18)
Change control	Drift in AI behavior; untracked model, prompt, or workflow changes	Version tracking for model, prompt, template, and workflow changes; visible review of material documentation changes at sign-off; CDI-mediated changes routed through compliant clarification processes	CDI leadership and HIM, jointly with clinical informatics	ACDIS/AHIMA Guidelines for Achieving a Compliant Query Practice (19); Joint Commission Quick Safety Issue 10 (15)
Monitoring	Undetected addition, loss, propagation; vendor model drift; clinician deskilling	Sampled prebill review with structured error categorization; tracking of unsupported additions, omissions, and propagation patterns by workflow or service line; version-aware monitoring of vendor updates; predefined escalation thresholds when error patterns exceed acceptable limits	Quality and CDI, with informatics analytics support	NIST AI 600-1 Manage and Measure functions (20); Health AI Partnership AI Vendor Disclosure Framework (21)
Auditability	Inability to reconstruct what AI produced, what clinician changed, what coder relied on	Retained audit trail of AI-generated drafts, edits, attestations, and available evidence links, with access for internal compliance review and external audit where required	Compliance and HIM	CMS Complying with Medical Record Documentation Requirements (MLN909160, 2024) (13); AHIMA prospective CDI standards (18)

The foundation is physician-led governance and oversight. Physicians do not need to perform every workflow task, but they should help define evidence thresholds, acceptable-use boundaries, and decision rights where AI changes clinical meaning rather than merely reformatting text. In AI-enabled documentation, governance cannot be reduced to vendor assurance or technical performance dashboards alone.

The second component is evidence visibility. Reviewers should be able to see not only the generated statement, but also the source context or evidence path on which it rests, especially for diagnoses, severity modifiers, and summarized care trajectories. If a reviewer cannot tell why a statement is present, the workflow is asking humans to validate outputs without adequate provenance. Recent work on retrieval-augmented fact verification of LLM-generated patient care documents has demonstrated that source-anchored verification is technically achievable (6).

Change control and configuration management form the third governance component. Prompts, templates, thresholds, workflow placement, and model versions all alter system behavior. Each requires local review, version tracking, and escalation pathways. NIST's Generative AI Profile is useful here because it reinforces lifecycle-based risk management rather than one-time pre-deployment assurance (20).

Monitoring and quality signals form the fourth component. Candidate measures include unsupported-statement rate, clinically relevant omission rate, major-correction rate after clinician review, and the proportion of AI-suggested changes overturned or narrowed during CDI or coding review. For example, organizations could periodically sample finalized encounters and have a clinician or CDI specialist adjudicate discrete factual statements in AI-shaped documentation as supported, unsupported, or indeterminate. The unsupported-statement rate would equal unsupported statements divided by statements adjudicated as supported or unsupported, with indeterminate statements reported separately. Results should identify the sampling frame and adjudicator role. Exceeding a predefined acceptable limit or showing a sustained increase from a validated baseline would trigger escalation, configuration review, narrowed scope, or a pause. Other measures can be specified using the same structure.

Monitoring should also include periodic unassisted assessment to detect loss of independent review capability, consistent with evidence on automation bias and the limits of clinician vigilance (7, 9). Clinicians or reviewers could periodically assess matched standardized cases with and without AI support, measuring detection of clinically significant documentation issues, false-positive rate, time to decision, and inter-rater agreement. The purpose is workflow-level capability surveillance, not individual performance management. A sustained decline in unassisted performance would trigger training, workflow redesign, or narrower automation.

Auditability and traceability close the framework. Record-shaping outputs should be attributable to a workflow state, model or configuration state, and review path. That attribution is what makes governance reviewable by internal compliance, external audit, and the clinicians and CDI professionals accountable for the record. Without it, drift becomes visible only after harm or rework.

A minimum viable implementation can begin small: one high-risk inpatient workflow using one AI documentation or coding-adjacent tool. Auditing a sampled prebill cohort against the three error modes above establishes the operational triggers for pausing or reconfiguring the workflow. Evaluation of such a pilot would track two signals. The primary signal is the compliant query rate at the prebill interval: if the framework is surfacing clarification opportunities that routine review misses, appropriately supportable queries should rise, and a sustained rise should prompt evaluation of upstream generation or workflow placement. The secondary, lagging signal is the rate of downstream corrections and denials at post-bill audit, tracked separately for documentation-level amendments and coding-level changes. These should decline as issues are resolved prospectively rather than discovered retrospectively. A null result on both signals would indicate that governance effort is better directed elsewhere for that workflow.

The control is not costless. It requires trained review capacity, analytics, and escalation pathways, and may add latency before claim submission. Depending on the implementation model, these costs may be distributed across CDI, HIM, coding, quality, compliance, informatics, contracted review services, and vendors; they should be explicitly allocated. A control plane need not require universal manual review. It may combine automated provenance checks, sampled monitoring, risk-tiered routing, and targeted expert adjudication, with scope that widens or narrows according to observed error rates, potential consequence, and available organizational capacity.

The real baseline is not zero governance but the cost of ungoverned drift — the constant friction of post-bill corrections, denial rework, and retrospective amendments, which may require more staff time per case and carry compliance exposure that earlier intervention avoids. The actual magnitude of that avoided cost relative to control-plane staffing is an empirical question this Perspective does not resolve, but it is the comparison future evaluation should target.

## Decision rights and shared responsibilities

Vendor disclosure frameworks improve procurement questions about intended use, performance, integration, and lifecycle stewardship (21, 24), but do not replace local governance over deployment, review, escalation, monitoring, and stop rules. Vendors remain responsible for accurate capability disclosure, performance within intended-use boundaries, version and change notification, and defect reporting. Health systems remain responsible for workflow placement, acceptance thresholds, and decisions to pause or reconfigure use. These responsibilities intersect at clinician sign-off, CDI prioritization, documentation nudges, and autonomous coding. Autonomous coding warrants distinct governance because it may reduce or remove routine coder review before claim submission.

Clinicians, CDI teams, and coders should retain explicit, differentiated decision rights over clinical statements, documentation clarification, and coded outputs. These functions should not be flattened into a generic human-in-the-loop claim. A clinician attesting to a note, a CDI reviewer evaluating a query opportunity, and a coder assigning a diagnosis code make different judgments against different evidence standards and accountability consequences. Governance that treats them as interchangeable miscounts both risk and capability.

Recent literature has argued that structural design, not clinician vigilance alone, must carry more of the AI safety load (9). This matters because documentation-governance interventions are not bedside clinical decision support. Their object is record integrity and translation, not immediate therapeutic action, and they therefore require different evidence standards, review pathways, and stop rules.

## Discussion: safer systems for patient safety, clinician safety, and record integrity

If health systems are going to scale AI-enabled documentation, governance should be evaluated against three safety goals: patient safety, clinician safety, and record integrity. This framing recognizes that AI documentation risk extends beyond immediate patient harm to clinician burden, liability exposure, and degradation of the medical record as the basis for care, coding, payment, and reporting.

Patient safety is implicated because misleading additions, meaningful omissions, or propagated distortions can alter later interpretation of the chart (4, 5, 15, 16).

Clinician safety is implicated because burden does not disappear when drafting becomes easier. It shifts. The system may reduce documentation work while increasing vigilance demands and liability exposure for clinicians expected to approve plausible but imperfect output. Current evidence supports real burden reduction but does not support treating clinician review as a frictionless safeguard (1–3). Recent work on factual verification of patient care documents and automation bias in AI-assisted reasoning strengthens that point by suggesting that subtle inaccuracies can be difficult to detect reliably and that even AI-literate physicians may be influenced by erroneous model output (6, 7, 9). Consistent with these concerns, a VHA-led evaluation of 11 ambient AI scribe tools using standardized primary care encounters found lower documentation-quality scores for AI-generated than clinician-produced notes across all measured domains, supporting continued review and editing of generated drafts (25).

In inpatient practice, AI-driven documentation prompts may appear alongside clinical decision support within the same workflow, and sometimes the same screen. Attention is finite. Clinicians may be asked to validate AI-shaped documentation, respond to documentation-improvement nudges, and act on clinical alerts while managing an acutely ill patient. In that convergence, clinical care must take priority over documentation vigilance. A safer system makes that hierarchy explicit. It does not rely on *ad hoc* clinician review at the point of conflict, but instead concentrates documentation review at moments when it is feasible, creates escalation paths for cases that exceed routine review capacity, and acknowledges that the highest-stakes documentation moments are often the moments least suited to careful clinician validation.

Record integrity is implicated because the medical record remains the authoritative narrative and evidentiary base on which coding, payment, organizational quality reporting, and future clinical reasoning depend. The prebill control plane is one way to address this. It is the governance layer designed to catch what direct and indirect AI shaping may have quietly altered before the record becomes operationally binding. The goal is therefore to reduce administrative burden without asking users to silently absorb unmeasured integrity risk.

TRIAD similarly frames intelligence as a property of the human-AI team and links trustworthy governance with real-world clinical value and adaptive deployment (26). The prebill control plane is complementary but narrower: TRIAD addresses clinical AI deployment broadly, whereas this framework specifies governance at the claim-linked interval where documentation, clarification, and coding converge.

## Limitations and scope

This Perspective has several limitations. First, the prebill control plane is conceptual. It does not claim that a prebill control plane has been empirically proven to improve documentation accuracy, denial rates, clinician burden, or patient-safety outcomes. Rather, it proposes a governance model for an operational interval in which AI-shaped documentation and coding-adjacent outputs are already entering claim-linked workflows. The model should be evaluated prospectively as inpatient evidence matures.

Second, the inpatient evidence base is growing but uneven. Hospital-only studies now support feasibility for discharge-summary generation, hospital-course summarization, patient-facing discharge transformation, and coding assistance under human review, but direct evidence remains limited for live inpatient bedside ambient-scribe workflows, autonomous coding, denial outcomes, billing integrity, clinician time, and patient-safety effects under production conditions (10–12). The framework proposed here is designed for inpatient coded-claims workflows, where care complexity, handoff density, documentation clarification, and coding consequences may differ materially across settings. Future work should test whether a prebill control plane improves documentation fidelity, clinician vigilance burden, denial risk, and downstream patient-safety or reporting outcomes in real inpatient settings.

Third, applying recursive model-collapse research to clinical documentation remains analogical rather than empirically established (17).

Fourth, the framework assumes organizational resources typical of large academic and tertiary care health systems — established CDI programs, clinical informatics capacity, HIM and compliance functions, and analytics infrastructure. Smaller community, rural, and resource-constrained organizations may need adapted implementation pathways, including shared services, pooled CDI resources, or phased adoption prioritizing evidence visibility and sampled prebill review before full monitoring infrastructure is feasible.

Fifth, the framework is grounded in United States inpatient structures, including CMS documentation requirements, DRG-based claims, the CDI profession, and AHIMA's definition of the prebill interval. Its broader applicability depends on whether another health system has a comparable point at which documentation can still be reviewed against recoverable context before coding, reimbursement, reporting, or legal finalization. Translation would therefore require mapping the relevant control interval, accountable roles, coding system, and regulatory obligations. Where no comparable prebill interval exists, the monitoring and auditability components may still apply at documentation or coding finalization.

## Conclusion

As AI-enabled documentation tools enter mid-revenue-cycle workflows, adoption should proceed with explicit governance and ongoing evaluation (1–3). The central informatics question is whether health systems can preserve a clinically faithful, auditable record as AI-generated content moves from encounter narrative to billable form.

The prebill interval is not a magic boundary. It is a practical one. It is where direct and indirect influences shape record integrity while source context is often still recoverable. A physician-led prebill control plane offers one workable governance response.

The task now is not merely to keep a human in the loop. In practice, that phrase can place invisible safety labor on clinicians while leaving the workflow itself under-governed. What AI-enabled documentation requires is a safer system for record integrity, built on physician-led governance, evidence visibility, change control, monitoring, and auditability. With explicit decision rights for clinicians, CDI teams, and coders, the prebill control plane is one way to pursue patient safety, clinician safety, and record integrity by supporting the practitioner rather than quietly transferring risk to them.

## Data Availability

The original contributions presented in the study are included in the article; further inquiries can be directed to the corresponding author.
